# Prexasertib, a Chk1/Chk2 inhibitor, increases the effectiveness of conventional therapy in B-/T- cell progenitor acute lymphoblastic leukemia

**DOI:** 10.18632/oncotarget.10535

**Published:** 2016-07-11

**Authors:** Andrea Ghelli Luserna Di Rorà, Ilaria Iacobucci, Enrica Imbrogno, Cristina Papayannidis, Enrico Derenzini, Anna Ferrari, Viviana Guadagnuolo, Valentina Robustelli, Sarah Parisi, Chiara Sartor, Maria Chiara Abbenante, Stefania Paolini, Giovanni Martinelli

**Affiliations:** ^1^ Institute of Hematology “L. e A. Seragnoli”, Department of Experimental, Diagnostic and Specialty Medicine, University of Bologna, Bologna, Italy

**Keywords:** CHK1, cell cycle, acute lymphoblastic leukemia, DNA damage response, chemo-sensitizer agent

## Abstract

During the last few years many Checkpoint kinase 1/2 (Chk1/Chk2) inhibitors have been developed for the treatment of different type of cancers. In this study we evaluated the efficacy of the Chk 1/2 inhibitor prexasertib mesylate monohydrate in B-/T- cell progenitor acute lymphoblastic leukemia (ALL) as single agent and in combination with other drugs. The prexasertib reduced the cell viability in a dose and time dependent manner in all the treated cell lines. The cytotoxic activity was confirmed by the increment of apoptotic cells (Annexin V/Propidium Iodide staining), by the increase of γH2A.X protein expression and by the activation of different apoptotic markers (Parp-1 and pro-Caspase3 cleavage). Furthermore, the inhibition of Chk1 changed the cell cycle profile. In order to evaluate the chemo-sensitizer activity of the compound, different cell lines were treated for 24 and 48 hours with prexasertib in combination with other drugs (imatinib, dasatinib and clofarabine). The results from cell line models were strengthened in primary leukemic blasts isolated from peripheral blood of adult acute lymphoblastic leukemia patients. In this study we highlighted the mechanism of action and the effectiveness of prexasertib as single agent or in combination with other conventional drugs like imatinib, dasatinib and clofarabine in the treatment of B-/T-ALL.

## INTRODUCTION

Nowadays the efficacy of conventional treatments for adult ALL is still very poor and several leukemia subtypes, like the once with complex karyotype or with Mixed Lineage Leukemia (MLL) rearrangement, continue to have a very poor prognosis due to not adequate treatment [1]. Moreover a large percentage of patients initially successfully treated, like the Philadelphia-positive ALL patients treated with tyrosine kinase inhibitors (TKIs) [2, 3], frequently develop resistance to treatment and consequently relapses [4]. Thus, there is a need to improve the therapeutic approaches for ALL adult patients. Recently, several international research groups have focused their studies on the potential effectiveness of the inhibition of specific kinases involved in the regulation of the cell cycle and in the DNA damage response (DDR) pathway [5–7]. In eukaryotic cells, the cell cycle is finely regulated by three different checkpoints (G1/S, intra-S and G2/M checkpoints) that control the transition from a specific phase to another [8]. The G1/S checkpoint is mainly controlled by the tumor-suppressor p53 [9] that regulates the transition through the G1 phase by enhancing the transcription of p21Waf1/Cip1 protein, a cyclin-dependent kinase (Cdk) inhibitors [10]. The transition through the S and the G2/M phase is regulated by the Chk1 and, at a lower rate, by the Chk2. Chk1 and Chk2 are two serine-threonine kinases involved in the response to DNA damages and, in particular, in the response to Single Strand Breaks (SSBs) and Double Strand Breaks (DSBs) of the DNA [11]. Numerous stimuli, like Ionizing Radiation (IR), X-ray exposure, replicative stress and chemotherapy drugs, activate the Chk1/Chk2 response [12]. Chk1 and Chk2 are activated, via phosphorylation, respectively by ataxia telangiectasia and Rad3-related (ATR) and by ataxia telangiectasia mutated protein (ATM) [13–15]. As a consequence of their activation, both Chk1/Chk2 arrest the cell cycle, activate DNA damages repair or promote cell death via apoptosis if the DNA damage cannot be resolved. Although no hereditary mutations of Chk1 or Chk2 have been found to promote tumor transformation, in numerous types of cancer the expression of these kinases have been found altered. In particular in several kinds of tumors the over-expression of Chk1 has been related to the resistance to treatments and to the increase of genetic instability. Due to their biological relevance, numerous Checkpoint Kinases inhibitors (Chk-i) have been developed in order to potentiate the efficacy of different antineoplastic drugs and to increase the cytotoxicity of radiotherapy [16–24]. Currently although the amount of publications and the number of clinical trials regarding the evaluation of the efficacy of Chk-i in solid tumor is in constantly growth [19, 20], only few studies have been done to demonstrate their efficacy in the treatment of hematologic malignancies [21] and even a lower number specifically on ALL [22]. Our group has recently showed the effectiveness of PF-0477736, a Chk1/Chk2 inhibitor, in single agent on a panel of different B-/T-ALL cell lines and on primary leukemic cells isolated from adult B-ALL patients. The results of the study showed that Chk1 kinase, but not Chk2, was significantly over-expressed in ALL patients in comparison to normal mononuclear cells and that the inhibition of Chk1/Chk2 reduced the cell viability and induced apoptosis on primary cells and several cell lines [22]. Starting from this background, in this study, we evaluated the *in vitro* efficacy of prexasertib mesylate monohydrate (hereafter referred to prexasertib), a novel Chk1/Chk2 inhibitor, in B- and T-progenitor ALL as single agent or in combination with different drugs like TKIs and other chemotherapy drugs like purine nucleoside analogue clofarabine. The prexasertib is a small molecule that acts as a selective ATP competitor inhibitor of Chk1 and Chk2 [25] proteins. Recently, the *in vitro/in vivo* effectiveness of the compound as a chemo sensitizer agent was assessed on different kinds of tumor models [26]. Nowadays this molecule is part of a clinical phase I study in patients with advance cancer as single agent, NCT01115790, and in combination with other chemotherapy drugs or radiotherapy (NCT02124148, NCT02555644). The chemo-sensitizer activity of the compound was evaluated combining prexasertib with different drugs normally used in the clinic of adult ALL patients [27]. In particular Philadelphia-positive ALL cell lines and primary leukemic cells were treated combining prexasertib with two TKIs (imatinib and dasatinib). The efficacy of TKIs in single agent or in combination with conventional chemotherapy have been well established for the treatment of ALL harboring the fusion protein BCR-ABL1 [28]. Although recently novel specific therapies have been tested for the treatment of Philadelphia-negative patients, most of them are still based on conventional chemotherapy. Today is crucial to develop therapeutic combinations that can increase the effectiveness and, simultaneously, reduce the side effects of conventional chemotherapies. For this reason Philadelphia-negative cell lines and primary cells were treated with prexasertib and with the 2'-deoxyadenosine analogue, clofarabine. Clofarabine has been showed to induce cell apoptosis due to the reduction of nucleoside triphosphate and consequently due to the inhibition of ribonucleotide reductase and DNA polymerases [29, 30].

## RESULTS

### Prexasertib inhibits cell viability in B-/T-ALL cell lines

**Figure 1:**
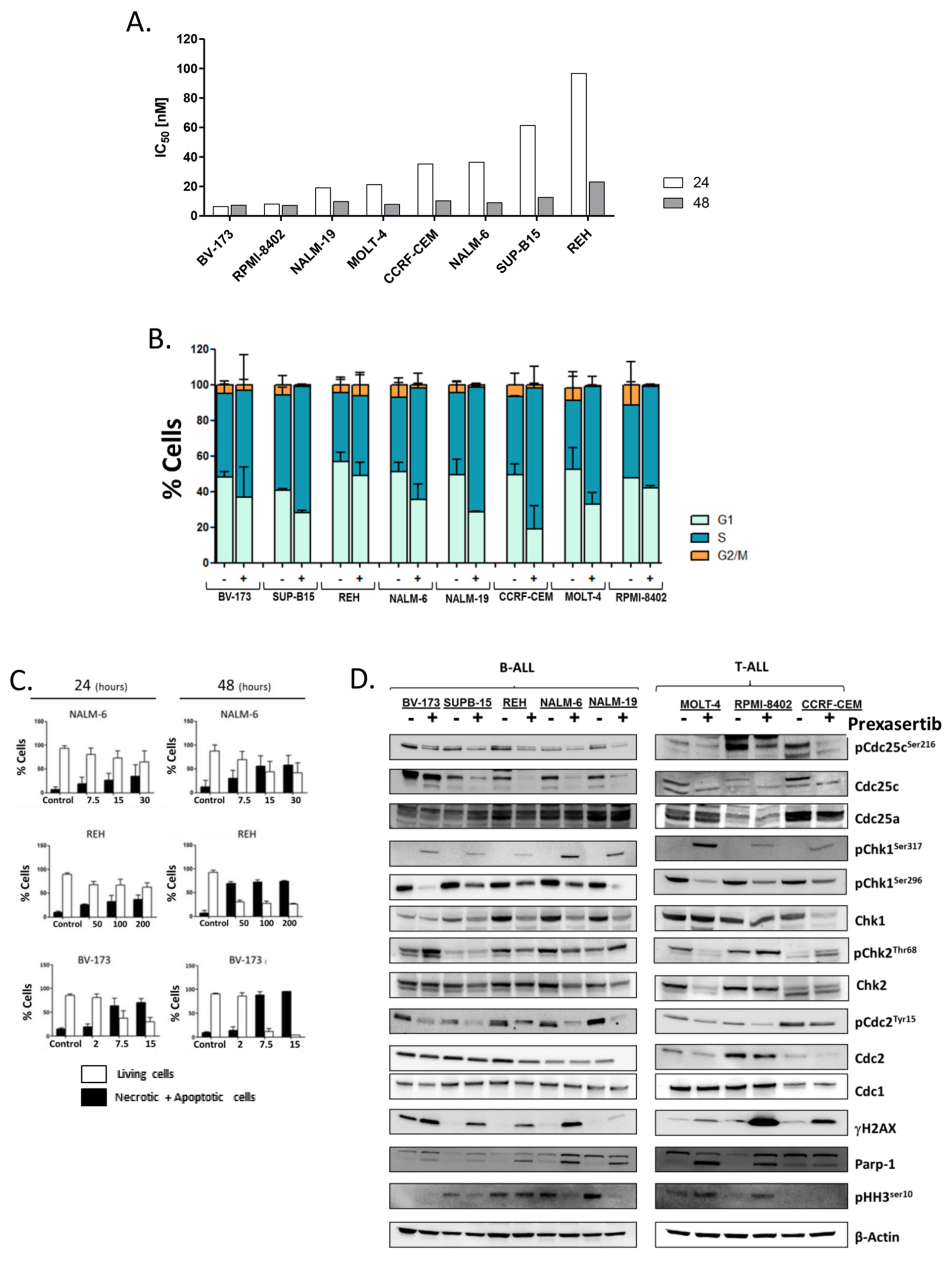
Effect of prexasertib on cell viability, induction of apoptosis, inhibition of Chk1 pathway and cell cycle profile in B-/T-ALL cell lines Graphical representation of the IC_50_ values of the B-/T-ALL cell lines after 24 and 48 hours of incubation with prexasertib. The IC_50_ values were obtained from two independent experiments **A.** Cell cycle profile of B-/T-ALL cell lines treated with or without prexasertib (IC_50_ value) for 24 hours **B.** Graphical representation of apoptosis induction by prexasertib. BV-173, NALM-6 and REH cells were treated with increasing concentration of drug for 24 and 48 hours **C.** The blots show, for each cell lines, the expression of key elements of the Chk1 pathway after 24 hours of incubation with prexasertib (IC_50_ value) **D.** In the figure the samples named Control were cells treated with 0.1 % of DMSO. In the Western blot analysis the homogeneity of the protein loaded (30 μg) was determined by using an internal control (β-actin).

The efficacy of the compound, in term of reduction of the cell viability, was firstly evaluated on a panel of different B-/T-ALL cell lines (BV-173, SUP-B15, REH, NALM-6, NALM-19, MOLT-4, RPMI-8402 and CCRF-CEM). In order to evaluate the cytotoxicity of the compound, the cell lines were incubated for 24 and 48 hours with increasing concentration of prexasertib (1-100 nM). The compound reduced the cell viability in all the treated cells in a time and dosage-dependent manner. Using specific statistical analysis, the IC_50_ values were detected for all the cell lines highlighting the BV-173 as the most sensitive cell line (6.33 nM) and the REH as the less sensitive one (96.7 nM). The sensitivity to the compound as single agent did not correlate with leukemia cell type (B-ALL *vs* T-ALL), with the mutational status of the tumor-suppressor p53 (BV-173, SUPB-15, NALM-6 and NALM-19 cells are p53 wild-type whereas REH, MOLT-4, RPMI-8402 and CEM cells are p53 mutated) (Figure 1A; Table 1) or with the basal expression of Chk1 or Chk2 proteins (data not showed). The correlation between the mutational status of p53 and the sensitivity to the compound was evaluated because of its role in the regulation of the G1-S checkpoint and in the response of DNA damages [38, 39].

**Table 1:** Leukemia sub-type, karyotype, mutational status of p53 and IC_50_ value (after 24 hours) of the panel of B-/T-ALL cell lines

CELL LINE	LEUKEMIA SUBTYPE	KARYOTYPE*	MUTATIONAL STATUS OF *TP53*	Prexasertib IC_50_**(nM)**
REH	PH-NEG. B-ALL	46(44-47)<2n>X, -X, +16, del(3)(p22), t(4;12;21;16)(q32;p13;q22;q24.3)-inv(12)(p13q22), t(5;12)(q31-q32;p12), der(16)t(16;21)(q24.3;q22) - sideline with inv(5)der(5)(p15q31),+18- carries t(12;21) and del(12) producing respective ETV6-RUNX1 (TEL-AML1) fusion and deletion of residual ETC6 (TEL)	MUT (R181C)	96.7
SUP-B15	PH-POS. B-ALL	46<2n>XY, der(1)t(1;1)(p11;q31), add(3)(q2?7), der(4)t(1;4)(p11;q35), t(9;22)(q34;q11), add(10)(q25), ?del(14)(q23q31), der(16)t(9;16)(q11;p13)	WT	61.4
NALM-6	PH-NEG. B-ALL	46(43-47)<2n>XY, t(5;12)(q33.2;p13.2) leading to ETV6/PDGFRB	WT	36.4
CCRF-CEM	T-ALL	90(88-101)<4n>XX, -X, -X, +20, +20, t(8;9)(p11;p24)x2, der(9)del(9)(p21-22)del(9)(q11q13-21)x2 - sideline with +5, +21, add(13)(q3?3), del(16)(q12)	MUT(R175H,R248Q)	35.3
MOLT-4	T-ALL	89-99<4n>XXYY, +4, +7, +8, +20, +20, del(6)(q16)x2, der(7)t(7;7)(p15;q11)x2	MUT(R248Q)	21.2
NALM-19	PH-NEG. B-ALL	47(45-48)<2n>XY,+5, del(9)(p21.2)	WT	19.1
REPMI-8402	T-ALL	90(79-91)<4n>XXX, -X, +3, +3, -10, -13, -14, +15, -18, -20, +2mar, dup(4)(q13q23)x2, del(6)(q14q22)x2, t(11;14)(p15;q11)x2, add(15)(p13) - sideline with der(1)t(1;9)(p35/36;q11), add(13)(q34) - carries t(11;14) with LMO1-TRD@ (LMO1-TCRD) rearrangement and cryptic del(1)(p32) effecting STIL-TAL1 (SIL-TAL1) fusion	MUT(R273C)	8.07
BV-173	PH-POS. B-ALL	47(46-48)<2n>X/XY, -9, +22, +mar, add(1)(q42), add(8)(p23), t(9;22)(q34;q11), der(22)t(9;22)(q34;q11), der(?)t(9;?)(?p11;?)	WT	6.33

* Leibniz-Institut DSMZ-Deutsche Sammlung von Mikroorganismen und Zellkulturen GmbH (Germany), www.dsmz.de

Abbreviations: Ph, Philadelphia; neg, negative; pos, positive; WT, wild-type; MUT, mutated.

### Prexasertib modifies the cell cycle profile in B-/T-ALL cell lines

In order to evaluate the effects of prexasertib on cell cycle progression, different cell cycle analyses were performed. Firstly, cells were treated for 24 hours using the doses nearest to the IC_50_ values and then stained for 1 hour with Propidium Iodide (Pi). In agreement with the study of King C. and colleagues [26] the treatment with prexasertib reduced the amount of cells in G1 and G2/M phase while increased the number of cells in S phase. In particular, the treatment statistically significant increased the number of cells in S phase in SUP-B15 (P-value 0.0338), NALM-6 (P-value 0.0421), NALM-19 (P-value 0.0282), CCRF-CEM (P-value 0.0253), MOLT-4 (P-value 0.0364) and RPMI-8402 (P-value 0.0379) cell lines but not in the most sensitive (BV-173: P-value, 0.4616) and in the less sensitive (REH: P-value, 0.6424) cell lines (Figure 1B). Secondly, the effect of prexasertib on cell cycle progression was analyzed treating ALL cell lines for different time points. In particular, BV-173, NALM-6 and REH cell lines were treated with prexasertib (respectively, with 7.5, 30 and 100 nM) for 18, 24, 30 and 48 hours and then stained with Pi. In BV-173 and REH cell lines even after 48 hours of incubation the amount of cells in S phase was not increased by the treatment, on contrary in NALM-6 cell line the amount of cell in S phase was increased in a time-dependent manner (Supplementary Figure S1A).

### Prexasertib as single agent activates the apoptotic cascade and targets the Chk1 pathway in B-/T-ALL cell lines

In order to better investigate the mechanism of action of prexasertib and to correlate the inhibition of cell viability with the induction of cell death, different Annexin V/Propidium iodide (Pi) staining analyses were performed. Cells were treated for 24 and 48 hours with increasing concentration of prexasertib (BV-173 and RPMI-8402: 2, 7.5 and 15 nM; NALM-6, NALM-19 and MOLT-4: 7.5, 15 and 30 nM; SUP-B15, REH and CCRF-CEM: 50, 100 and 200 nM) and the number of apoptotic/necrotic cells was evaluated using cytofluorimetry. In line with the results from the cell viability assays, the treatment enhanced cell death via apoptosis in a time and dosage-dependent manner (Figure 1C). The activation of the apoptotic cascade was confirmed by immunoblotting as showed by the cleavage of the apoptotic marker, poly (ADP-ribose) polymerase 1 (PARP-1) (Figure 1D). Successively, the results found on the cell cycle profile analyses and on the apoptosis analyses were correlated with the modifications of the expression of different proteins involved in the DDR pathway. To this purpose, cells were treated with prexasertib (doses nearest to IC_50_ values) for 24 hours and then stained for different markers. First of all, to evaluate the on-target activity of the compound, samples were stained for phospho-Chk1^ser296^, marker of inhibition of the functionality of Chk1. In all samples the treatment reduced the amount of phospho-Chk1^ser296^ confirming that Chk1 was specifically inhibited by prexasertib. The treatment with prexasertib modified the expression of different downstream targets of the Chk1/Chk2 pathway most of them involved in the regulation of the G2/M checkpoint. In particular, the alteration of the number of cells in G2/M phase seen in the cell cycle analysis was confirmed by the reduction of both basal and phosphorylated isoforms of the phosphatase CDC25C (phospho-CDC25C^ser216^) and of the kinase cyclin-dependent, CDC2 (phospho-CDC2^Tyr15^). No significant changes have been found on the amount of the basal forms of both CDC25A and CDC1. To investigate the effect of prexasertib in the induction of DNA damages, cells were stained for the following antibodies: phospho-Chk1 ^ser317^ (marker of activation of the Chk1 pathway), phospho-Chk2^thr68^ (marker of activation of the Chk2 pathway), γ-H2A.X (marker of induction of DNA damages) and Parp-1 (marker of induction of cell death via apoptosis). In all the cell lines the induction of DNA damage after the treatment was confirmed by increment of both phospho-Chk1^ser317^ and γ-H2A.X, while the activation of apoptosis was confirmed by the cleavage of Parp-1. The treatment changed in a very heterogeneous manner the expression of both basal and phosphorylated form of the kinase Chk2 (phospho-Chk2^Thr68^). In particular phospho-Chk2^Thr68^ was increased in BV-173, NALM-19, RPMI-8402 and CCRF-CEM cell lines while decreased in REH, NALM-6, NALM-19 and MOLT-4. No significant changes have been seen on SUP-B15 cell lines after the treatment. The effect of the compound on the total amount of Chk2 was very heterogeneous among the different cell lines. In all the treated cell lines prexasertib reduced the total amount of Chk2, with the exclusion of BV-173 and REH cell lines in which the treatment did not modify the amount of Chk2. Finally in order to highlight if the increment of the DNA damages and the activation of the apoptosis could be related with the induction of cell death by mitotic catastrophe, the expression of the phosphorylated form of the histone H3 (phospho-HH3^ser10^), marker of mitosis, was evaluated. The expression of phospho-HH3^ser10^ was reduced in SUP-B15, REH, NALM-6 and NALM-19 while was increased in MOLT-4 and RPMI-8402 cell lines. In BV-173 and CCRF-CEM cell lines no significant level of phospho-HH3^ser10^ was detected (Figure 1D). The reduction of the amount of phospho-HH3^ser10^ on NALM-6 and REH cell lines was confirmed also by flow cytometry. Cells were treated for 18, 24, 30 and 48 hours and then co-stained using Pi with a primary conjugated antibody, phospho-HH3^ser10^ (FITC-conjugated). In line with the immunoblotting results, the treatment reduced the amount of cells positive for the pHH3^ser10^ in both the cell lines (Supplementary Figue S1B). The effect of prexasertib in term of protein expression was also analyzed at different time points. These experiments highlighted that the effects of Chk1 inhibition were time-dependent, especially in term of the induction of DNA damages (γ-H2A.X) (Figure S1C). The hypothetical mechanism of action of the compound in term of perturbation of cell cycle checkpoint functionality and in term of progressively accumulation of DNA damages is graphically showed in Figure 5.

### Prexasertib increases the cytotoxicity of different tyrosine kinase inhibitors (TKIs) in philadelphia-positive cell lines

**Figure 2:**
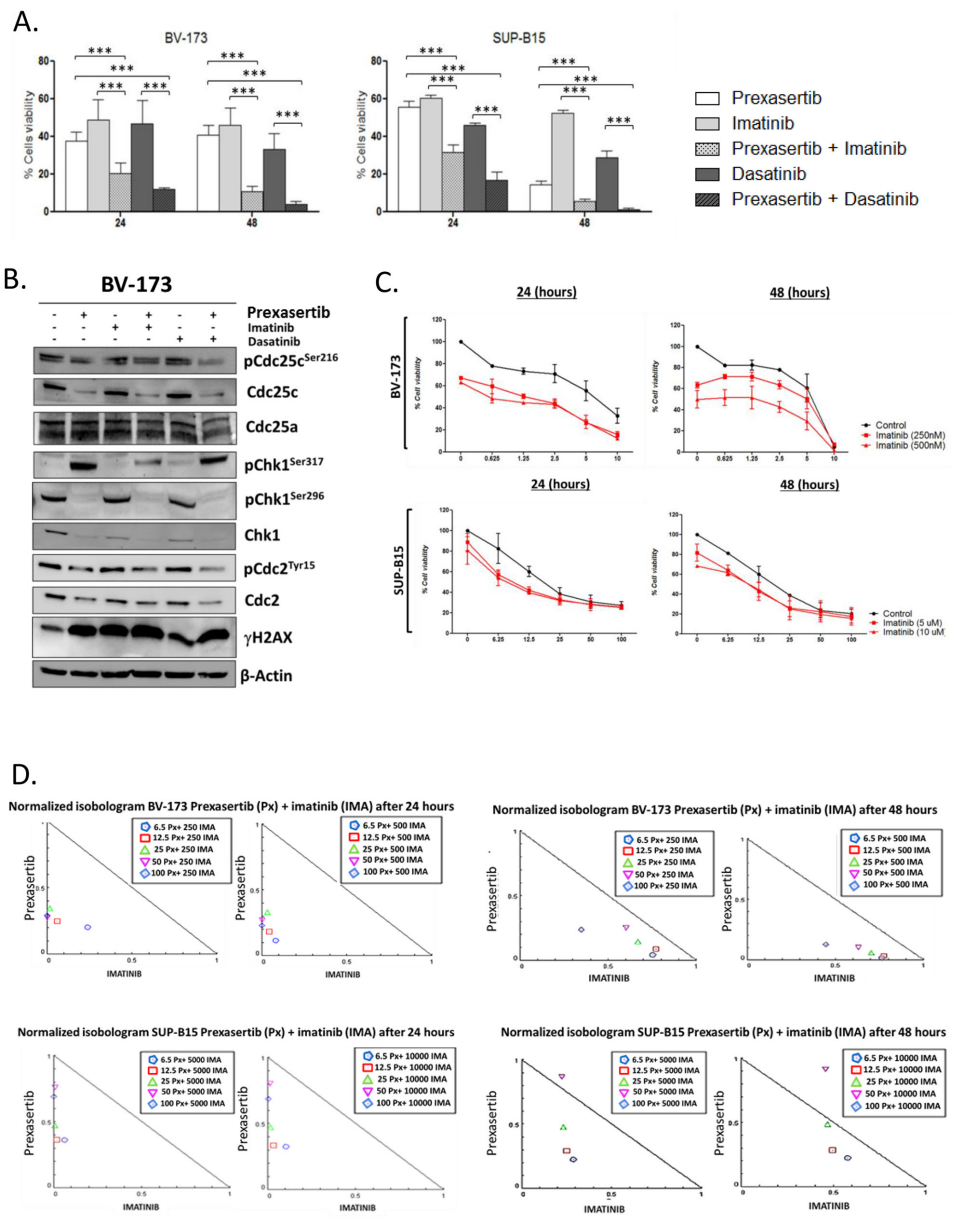
Effect of prexasertib in combination with TKIs in Philadelphia-positive cell lines Cell viability analysis of BV-173 and SUP-B15 cell lines incubated with prexasertib and TKIs (imatinib and dasatinib) for 24 and 48 hours. For each cell lines three different experiments have been performed **A.** The blot shows the expression different proteins of the Chk1 pathway after 24 hours of incubation with prexasertib in combination with the two TKIs. The cell line BV-173 was incubated with: prexasertib: 7nM; imatinib: 500nM; dasatinib: 50nM **B.** Combination index analysis of BV-173 and SUP-B15 cell lines incubated with increasing concentration of prexasertib (BV-173: from 10 to 0.6 nM, dilution rate 1:2; SUP-B15: from 100 to 6.25 nM, dilution rate 1:2) and two increasing concentrations of imatinib (BV-173: 250 and 500 nM; SUP-B15: 5 and 10 μM) for 24 and 48 hours. The black curve (control) represents the effect of the prexasertib alone while the two red curves represent the two combinations (full square prexasertib + 250 nM (BV-173) or +5 μM (SUP-B15) of imatinib; full triangle prexasertib + 500 nM (BV-173) or 10μM (SUP-B15) of imatinib). The curves represent the mean of two independent experiments **C.** Normalized Isobologram graphs represent the effect (Combination Index, C.I.) of the combination between prexasertib and imatinib on BV-173 and SUP-B15 cell lines after 24 and 48 hours of combination. Each combination is represented in the legend with a specific symbol. **D.** In the figure the samples named Control were cells treated with 0.1 % of DMSO. In the Western blot analysis the homogeneity of the protein loaded (30 μg) was determined by using an internal control (β-actin).

In order to assess the chemo-sensitizer ability of prexasertib, different experiments combining the Chk1/Chk2 inhibitor with different compounds routinely used in the clinic management of ALL were performed. To this purpose two Philadelphia-positive cell lines were treated for 24 and 48 hours with prexasertib (BV-173 7.5 nM; SUP-B15 50 nM) in combination with two different TKIs, imatinib (BV-173 500 nM; SUPB15 10 μM) and dasatinib (BV-173 50 nM; SUP-B15 500 nM), and the reduction of the cell viability was evaluated. In both treated cell lines the combinations increased the cytotoxicity of the two TKIs respects to the effect of the single treatments (Figure 2A). The consequences of the co-treatment, in term of change in the expression of different key elements of the Chk1 pathway, were investigated using Western blot. BV-173 cell line was incubated with prexasertib (7.5 nM) and with or without the two TKIs (imatinib: 500 nM; dasatinib: 50 nM) for 24 hours. The single treatments with the TKIs as well as their combinations with prexasertib did not significantly change the key elements of the Chk1 pathway. However in term of induction of DNA damages (γH2A.X) the co-treatment resulted in an additive effect in both the combinations (Figure 2B). Then to better understand the chemo-sensitizer activity of the compound and to evaluate the effect of the combination between prexasertib and TKIs using sub-toxic concentrations, different combination index assays were performed. BV-173 and SUP-B15 cell lines were incubated with increasing concentration of prexasertib (BV-173: from 0.6 to 10 nM, dilution rate 1:2; SUP-B15: from 6.25 to 100 nM, dilution rate 1:2) and two increasing concentration of imatinib (BV-173: 250 and 500 nM; SUP-B15: 5 and 10 μM) for 24 and 48 hours. To clarify the additive or synergic effect of the combinations, different isobologram analyses were performed. The results of these analyses confirmed the synergistic effect of the combination on both BV-173 and SUP-B15 cell lines after 24 hours of co-treatment (Combination Index = C.I. <1). The synergic effect of the combinations was confirmed also after 48 hours of co-treatment for BV-173 but not for SUP-B15 cell. Indeed in SUP-B15 cell line only the first three combinations showed an C.I. lower than 1 (Figure 2C-2D).

### Prexasertib increases the cytotoxicity of clofarabine in Philadelphia-negative cell lines

**Figure 3:**
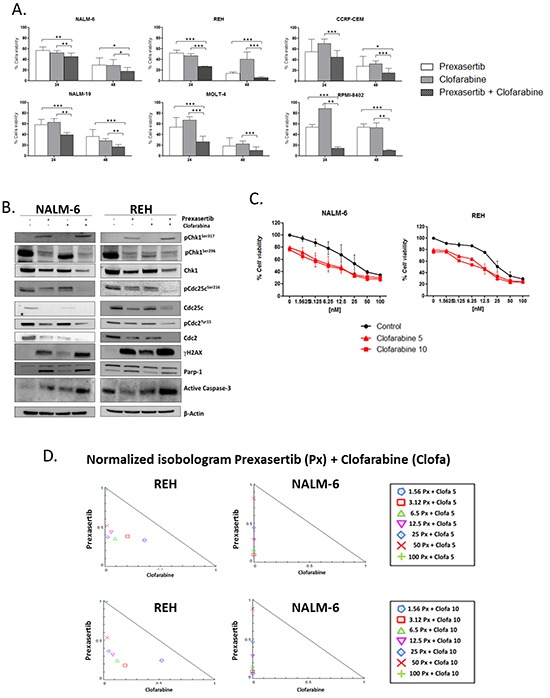
Effect of prexasertib in combination with clofarabine in Philadelphia-negative cell lines Cell viability analysis of Philadelphia-negative ALL cell lines incubated prexasertib and clofarabine for 24 and 48 hours. For each cell lines three independent experiments have been performed **A.** The blots show the expression on NALM-6 and REH cell lines of different proteins of the Chk1 pathway after 24 hours of treatment with prexasertib in combination with clofarabine. The dosages of each drug have been chosen based on the IC_50_ values after 24 hours of incubation **B.** Combination index assay of NALM-6 and REH cell lines treated with increasing concentration of prexasertib (from 1.5 to 100 nM) and sub-toxic concentration of clofarabine (5 and 10 nM). The black curve (Control) represents the effect of the prexasertib alone while the two red curves represent the effect of the combinations (full triangle prexasertib + 5 nM of clofarabine; full square prexasertib +10 nM of clofarabine) after 48 hours of incubation with the two drugs. The curves in graph are representative of the mean of two independent experiments **C.** Normalized Isobologram graphs represent the effect (Combination Index, C.I.) of the combination between prexasertib and clofarabine on NALM-6 and REH cell lines. Each combination is represented in the legend with a specific symbol **D.** In the figure the samples named Control were cells treated with 0.1 % of DMSO. In the Western blot analysis the homogeneity of the protein loaded (30 μg) was determined by using an internal control (β-actin).

The efficacy as chemo-sensitizer agent of the prexasertib on Philadelphia-negative B-/T-ALL cell lines was evaluated combining the Chk1/Chk2 inhibitor with the purine nucleoside antimetabolite clofarabine, which is commonly used in clinical trials for the treatment of young and adult ALL patients [31, 32]. The effect of clofarabine in term of reduction of the cell viability was evaluated using WST-1 reagent and for each cell lines was calculated the IC_50_ value (data not showed). The effect of the combination was very heterogeneous between the different cell lines (Figure 3A). Interestingly no significant differences in term of effectiveness of the combination was seen between cell lines with p53 mutated, that should have an impaired function of the G1/S checkpoint, and with p53 wild type (Table 1). To investigate the effect of the combination of these two drugs and to evaluate if the cytotoxic effect could be dependent on a particular schedule, different experiments were performed. For this reason NALM-6 and REH cell lines were treated respectively with clofarabine alone for 48 hours (50 nM), prexasertib alone for 48 hours (10 nM), pre-incubated with clofarabine for 24 hours and then with prexasertib for 24 hours or pre-incubated with prexasertib for 24 hours and then with clofarabine for 24 hours. No significant differences were found between the different schedules (Supplementary Figure S1C). As with the previous combinations with TKIs, the consequences of the combination on the Chk1 pathway were evaluated using Western blot analysis. REH and NALM-6 cell lines were incubated for 24 hours with the checkpoint inhibitor in combination with clofarabine using for each drug the dose nearest to the IC50 after 24 hours (clofarabine: 50 nM; prexasertib 100 and 30 nM, respectively). The treatment with clofarabine as single agent changed the expression of different elements of the Chk1 pathway and in particular, the total amount of CDC2, CDC25C as well as the phosphorylated form of Chk1^Ser296^ were decreased by the treatment while the amount of γ-H2A.X was increased. The combination of the two drugs, in both cell lines, additively changed the levels of expression of different proteins involved in the Chk1 pathway. In particular, the co-treatment increased the DNA damages (γ-H2A.X) and the induction of apoptosis (Parp-1 and pro-Caspase 3 cleavage) while decreased the level of expression of phospho-CDC25C^ser216^ and phospho-CDC2^tyr15^ when compared to the single drugs (Figure 3B). The ability of prexasertib to sensitize leukemic cell lines to the cytotoxicity of clofarabine was evaluated performing different combination index analyses. To this purpose NALM-6 and REH cell lines were incubated with increasing concentration of prexasertib (from 0 to 100 nM dilution rate 1:2) and with two different concentrations of clofarabine (sub-toxic concentration: 5 and 10 nM ) for 24 and 48 hours. In REH cell line, with the exclusion of the combination between prexasertib 100 nM and 5/10 nM of clofarabine in which the effect of the combination was additive (C.I.=1), the cell viability was synergistically reduced (C.I. <1) by the co-treatment. In NALM-6 cell lines the cell viability was synergistically reduced in all the combinations with the exclusion of two combinations (prexasertib 1.56nM and 5 nM of clofarabine; prexasertib 100 nM and 5/10 nM of clofarabine) in which the effect was antagonistic (C.I.>1) (Figure 3C, 3D).

### Prexasertib targets the Chk1 pathway on leukemic blasts but not on peripheral blood mononuclear cell of healthy donors

**Figure 4:**
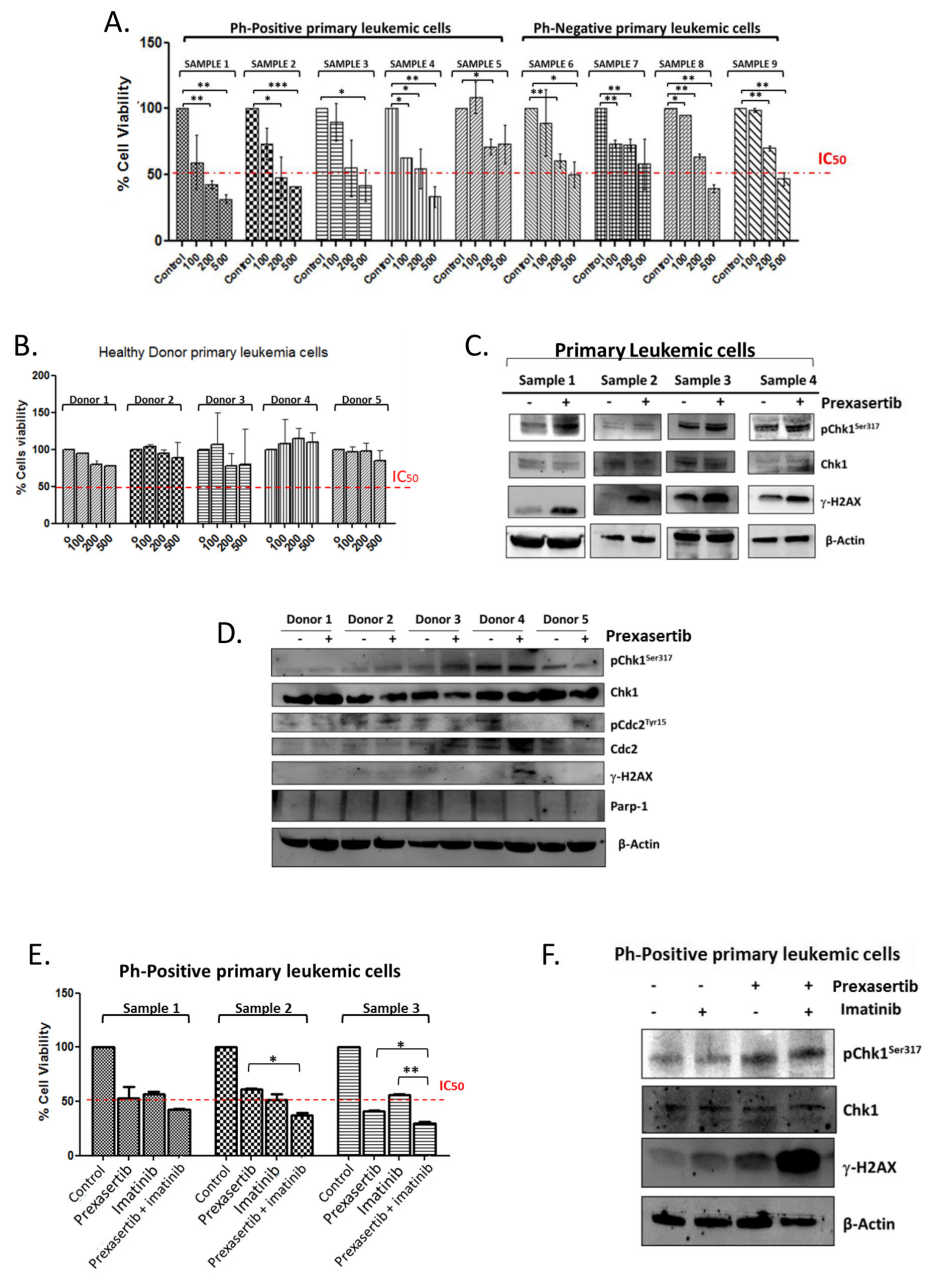
Effect of prexasertib on primary leukemic cells isolated from adult ALL patients and on peripheral mononuclear cells isolated from healthy donors Cell viability analysis on primary leukemia cells isolated from 9 adult ALL patients treated with increasing concentration of prexasertib (100, 200 and 500 nM) for 24 hours. **A.** Cell viability assay of mononuclear cells isolated form the peripheral blood of 5 healthy donors, treated with increasing concentration of prexasertib (100, 200 and 500 nM) **B.** The blot shows the induction of DNA damages on primary leukemic cells isolated from the peripheral blood of 4 newly diagnosed ALL patients treated for 24 hours with prexasertib (100nM) **C.** The blot shows the expression of different proteins of the Chk1 pathways after 24 hours of incubation with prexasertib (100nM) on the mononuclear cells isolated from the peripheral blood of 5 healthy donors **D.** Cell viability assay of primary leukemia cells isolated from 3 Philadelphia-positive ALL patients treated with prexasertib (100nM) and imatinib (5 μM) for 24 hours. The graph represents the main of response to the combination of the three patients **E.** The blot shows the induction of DNA damages on primary leukemia cells isolated from a Philadelphia-positive patient after 24 hours of treatment with prexasertib (100nM) in combination with imatinib (5μM) **F.** In the Western blot analysis the homogeneity of the protein loaded (30 μg) was determined by using an internal control (β-actin).

Finally, the effectiveness of the compound was evaluated on different primary leukemic cells isolated from the bone marrow and the peripheral blood from 9 adult B-ALL patients and on the mononuclear cells isolated from the peripheral blood from 5 healthy donors. To this purpose the primary cells were treated with increasing concentration of prexasertib (100, 200 and 500 nM) for 24 hours and then the reduction of the cell viability was evaluated using Trypan blue exclusion dye method. Similarly to the results found on B-/T-ALL cell lines, prexasertib progressively reduced the cell viability in a dose-dependent manner in all the primary leukemic cells (Figure 4A). However on the mononuclear cells isolated from the healthy donors the treatment did not significantly reduce the cell viability (Figure 4B). To correlate the reduction of cell viability with the induction of DNA damages, the primary cells isolated from the bone marrow of 4 adult B-ALL patients were treated with a sub-toxic concentration of the compound (100 nM) and then stained for phospho-Chk1^ser317^, Chk1 and γH2A.X. The Western blot analysis showed that in all samples, although with a high heterogeneity, prexasertib increased both markers of DNA damage (phospho-Chk1 ^ser317^and γH2A.X) (Figure 4C). By contrast, in normal cells the effect of the compound did not reduce significantly the cell viability and did not modifie the amount of the different downstream targets of Chk1 neither increased the amount of the phosphorylated form of γH2A.X, with the exclusion of one sample (Figure 4D). The chemo-sensitizer ability of prexasertib was then evaluated also on the primary leukemic cells. To assess that, the primary cells isolated from 3 Philadelphia-positive ALL patients were treated for 24 hours with prexasertib (200 nM) in combination with imatinib (5 μM). The concomitant treatment showed an additive effect in term of reduction of the cell viability in comparison with the effect of the single treatment (Figure 4E). Finally, to correlate the reduction of the cell viability to the induction of DNA damage, primary cells from one Philadelphia-positive ALL patient were treated with prexasertib (100nM) and imatinib (5uM) in combination and then analyzed by western blot. The co-treatment synergistically increased the amount of γH2A.X and additively increased the phosphorylated for of Chk1^ser317^ in comparison with the effects of the single treatments (Figure 4F).

## DISCUSSION

**Figure 5:**
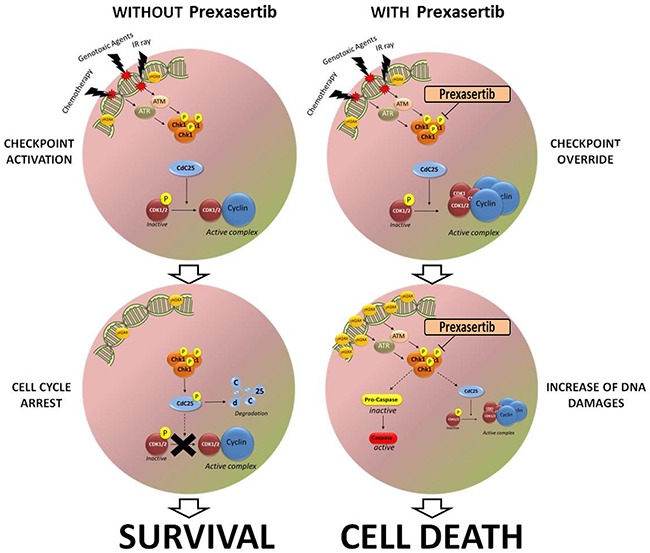
Schematic representation of the effect of prexasertib on leukemic blast after the exposure to different genotoxic agent The left side of the cartoon hypothesizes how leukemic cell could survive to chemotherapy drugs and to other genotoxic agent, activating the cell cycle checkpoint and arresting the cell cycle progression. The right side of the cartoon hypothesizes the mechanism of action of the compound in enhancing cell death and inducing checkpoint override after the exposure to different DNA damaging agents.

Nowadays, several studies have showed the effectiveness of different Chk1/Chk2 inhibitors as monotherapy or in combination with chemotherapy drugs or radiotherapy for the treatment of different kind of tumors [32–38]. The biological hypothesis of these studies is that tumor cells can survive to therapy activating the DDR and delay the cell cycle progression to prevent lethal cell division [20]. Many compounds arrest tumor cells in different phases of the cell cycle. For instance, the purine nucleoside analogue clofarabine, arrests tumor cells in G1/S phase due to the inhibition of DNA synthesis [39]. One of the most important regulators of the cell cycle is the Checkpoint kinase 1 (Chk1). In BCR/ABL1-positive cells the hyper activation of the ATR-Chk1 pathway after the exposure with different genotoxic agents has been associated with a delay in G2/M progression and thus with a possible mechanism of resistance to treatment [40]. Sarmento and colleagues found that T-ALL cells over-express Chk1 and then demonstrated that the aberrant expression is necessary for the proliferation and the survival of cancer cells [41]. In this study we demonstrated that Chk1 functionality is fundamental for the survival of B-/T-ALL cell lines and primary cells, and that the inhibition of this kinase using, prexasertib, sensitized both cell lines and primary cells to the cytotoxicity of different compound normally used for the treatment of adult B-/T-ALL patients. In line with our previous work evaluating the efficacy of the Chk1/Chk2 inhibitor, PF-0477736, the treatment with prexasertib reduced the cell viability in a time and dose-dependent manner in all the treated cell lines. The sensitivity to the compound was not correlated with the mutational status of p53, with the leukemia sub-type (Table 1) or with the basal expression of Chk1/Chk2 (data not shown). To investigate the effect of prexasertib on cell cycle regulation and to clarify mechanisms of cell death in different cell lines, cell cycle and western blots analyses were performed. In several B-/T-ALL cell lines the compound reduced the amount of cells G2/M phase and increased the percentage of cells in S phase. The reduction of the number of cells G2/M phase and the increase of cells in S phase together with heterogeneous modification of the mitotic marker phospho-Histone H3 (pHH3^ser10^) excluded the mechanism of cell death through mitotic catastrophe and corroborated the hypothesis of King C. and colleagues [26] of the *replication catastrophe*. However in the most sensitive, BV-173, and in the less sensitive, REH, cell lines the treatment with prexasertib induced DNA damages and reduced the cell viability independently to cell cycle modification highlighting other mechanisms of cell death that could be not connected neither to the *mitotic catastrophe* nor to the *replication catastrophe*. The single agent efficacy of prexasertib showed on the cell lines were confirmed on different primary leukemic cells isolated from adult B-ALL patients. Although the compound reduced the cell viability and increased the DNA damages in all the primary cell treated, we could not define any predictive factors of response. The idea of interfering with DNA damage response pathway in human cancer as a means to improve the cytotoxicity of DNA damaging therapies has been demonstrated in many pre-clinical studies. Here we evaluated the effectiveness of the prexasertib as a chemo-sensitizer agent for the treatment of ALL. We showed that prexasertib sensitized both Philadelphia-positive cell lines and primary leukemic cells to the cytotoxic effect of the two TKIs, imatinib and dasatinib. The combinations (prexasertib+TKIs) not only synergistically reduced the cell viability in comparison to the effect of the single treatments but also increased the amount DNA damages (γH2A.X), confirming the abrogation of the DNA damage response and the consequently accumulation of DNA damages. Similar results were found on different Philadephia-negative cell lines, combining prexasertib with the 2'-deoxyadenosine analogue, clofarabine. Finally, by assessing different combination schedules between prexasertib and clofarabine, we demonstrated that there was no significantly difference in term of reduction of the cell viability if the DNA damaging agent (clofarabine) was added before or after the DDR inhibitor (prexasertib). The results showed in this manuscript, in our opinion, are the basis for a future evaluation of this compound in clinical trials. We have already showed in our previous work [22] that Chk1 is over-expressed in ALL cells in comparison to the expression in normal tissue. Moreover the over-expression of this kinase is fundamental for ALL cells to sustain the high genetic instability and to proliferate. For this reason, we believe that the treatment with prexasertib could be a promising strategy to selectively kill cancer cells and preserve normal tissue. Moreover, the results of the combinatorial studies showed that the inhibition of the DNA damage response (DDR) pathway could be an innovative strategy to limit adverse events, by reducing the doses needed to reach a sufficient cytotoxic effect, and could increase the effectiveness of imatinib, dasatinib and clofarabine, by inhibiting the survival of cancer cells (Figure 5).

## MATERIALS AND METHODS

### Leukemia cell lines and primary samples

Human B- (BV-173, SUP-B15, REH, NALM-6, NALM-19) and T-ALL (MOLT-4, RPMI-8402, CCRF-CEM) cell lines were obtained from Leibniz-Institut DSMZ-Deutsche Sammlung von Mikroorganismen und Zellkulturen GmbH (Germany). Cells were cultured in RPMI-1640 medium (Invitrogen, Paisley, UK) with 1% *l*-glutamine (Sigma, St. Louis, MO), penicillin and streptomycin (Gibco, Paisley, UK) supplemented with 10%-20% fetal bovine serum (Gibco) in a humidified atmosphere of 5% CO_2_ at 37°C. Primary blast cells from 9 newly B-ALL cases were obtained, upon written informed consent, from the bone marrow and the peripheral blood by density gradient centrifugation over Lymphoprep (Nycomed UK, Birmingham). Mononuclear cells were isolated from the peripheral blood of 5 healthy donors. Online databases have been interrogated to molecularly characterize leukemia cell lines: International Agency for Research on Cancer (IARC) *TP53* database (http://www-p53.iarc.fr/) and the Catalogue of Somatic Mutations in Cancer, (COSMIC, http://www.sanger.ac.uk/genetics/CGP/cosmic/).

### Drugs and reagents

The Chk1/Chk2 inhibitor prexasertib was provided by Lilly Oncology. The purine nucleoside antimetabolite (clofarabine) and the tyrosine kinase inhibitors (imatinib and dasatinib) were bought by Sigma-Aldrich web site.

### Cell viability assay

To evaluate the cytotoxic effect of the treatment with prexasertib, B- and T-All precursor leukemia cell lines were seeded in 96-well plates at 50,000 cell/100 μl/well with increasing concentrations of drug (1-100 nM) for 24 and 48 and incubated at 37°C. Cell viability was assessed by adding WST-1 reagent (Roche Applied Science, Basel, Switzerland) to the culture medium at 1:10 dilution. Cells were incubated at 37°C and the optical density was measured by microplate ELISA reader at λ450 after 3 hours. The amount of the formazan formed directly correlates to the number of metabolically active cells. All viability experiments were performed in triplicates and repeated in least two separated experiments.

### Primary leukemic cells viability assay

To assess the effect of prexasertib on primary leukemic blasts, the cells from 9 newly diagnosed Philadelphia-positive and Philadelphia-negative ALL cases were obtained, upon written informed consent, from the peripheral and bone marrow blood samples by density gradient centrifugation over Lymphoprep (Nycomed UK, Birmingham).Primary cells were seeded on a 6 well plates at 500.000 cells/ml and incubated with increasing concentration of prexasertib for 24 hours at 37°C. In ex-vivo primary leukemia cells the effects on cell viability was assessed by counting viable and non-viable cell numbers by the Trypan blue dye exclusion method.

### Combination index assay

In order to evaluate the effectiveness of prexasertib as chemo-sensitizer agents, B-/T-ALL cell lines were seeded in 96-well plastes at 50,000 cell/100μl/well with increasing concentration of the compound (dilution rate 1:2) and increasing concentration of a second drug (dilution rate 1:2), for 24 and 48 hours at 37°C. Cell viability was assessed using WST-1 (Roche Applied Science, Basel, Switzerland). Cells were incubated at 37°C and the optical density was measured by microplate ELISA reader at λ450 after 3 hours. The amount of the formazan formed directly correlates to the number of metabolically active cells. All viability experiments were performed in triplicates and repeated in least two separated experiments. The additive, synergistic, and antagonistic effect of the combination was evaluated using Compusyn Software, using normalized isobologram graph and calculating the combination index, C.I.( C.I<1 synergism; C.I. =1 additivity; C.I.>1 antagonism).

### Annexin V staining of apoptotic cells

To assess the effect of the compound in the induction of apoptosis, three different increasing concentrations of prexasertib were used to treat cells lines in order to detect apoptotic, necrotic and cell debris. Cell lines were seeded in 12-well plates at 500,000 cell/1 ml with increasing concentrations of drug (NALM-6, NALM-19, MOLT-4 and CCRF-CEM: 7.5, 15 and 30 nM; BV-173 and RPMI8402: 2, 7.5 and 15 nM; SUP-B15 and REH: 50, 100 and 200 nM) for 24 and 48 hours and incubated at 37°C. Following the treatment, cells were harvested and stained with Annexin V/Propidium Iodide (PI) according to the manufacturer's instruction (Roche). The percentage of Annexin V-PI positive cells was determined within 1 × 10^4^ cells of the population by flow cytometry (Facs CantoII, BD Biosciences Pharmingen, San Jose, California, USA). The mean percentage of Annexin V-PI positive cells and standard error measurement was calculated from at least two separate experiments.

### Western blot analysis

All cell lines were plated in 6-well plates at 500,000 cells/1 ml with increasing concentrations of drug for different time points and incubated at 37°C. For evaluate the effect of combination of the Chk inhibitor with other drugs (imatinib, dasatinib and clofarabine) were used as dosage the IC_50_ for the single drug after 24 hours of treatment. The drugs in the combination were added simultaneously. At the end of the treatment, the cells were collected and lysate using a specific buffer made of KH2PO4 0,1 M (pH 7,5), Igepal 1% (NP-40), β-glicerofosfato 0,1 mM and complete protease inhibitor cocktail 1X (Roche Diagnostics). For each sample 30-50 μg of protein were fractioned on Mini-Protean TGX stain-free precasted gels, blotted to nitrocellulose membranes (Bio-Rad Trans-blot turbo transfer pack) and incubated overnight with the following antibodies: Chk1 (#2345S), phosphorylated Chk1 (Ser317)(#2344S), phosphorylated Chk1(Ser296)(#2349S) and Chk1 (Ser345)(#2348S), Cdc25c (#4688S), phosphorylated Cdc25C (Ser216)(#9528S), Cdc25a (#3652), Cdc2 (#9112S), phosphorylated Cdc2 (Tyr15)(#4539S), Chk2 (#2662S), phosphorylated Chk2 (Thr68)(#2661S), phosphorylated H2A.X (Ser139) (γ-H2A.X) (#2577S), Caspase3 (#9662S), Parp (#9542S),phosphorylated Histone 3 (Ser10) from Cell Signaling. Antibody for CDK2 (Cdc1)(sc-163) came from Santa Cruz biotechnology. Antibody to β-actin came from Sigma (St. Louis, MO). Finally all these antibodies were detected using the enhanced chemiluminescence kit ECL (GE) and the compact darkroom ChemiDoc-It (UVP).

### Cell cycle analysis

The cell lines were seeded in a 24 wells plate at the concentration of 500,000 cells/1 ml and treated for 6 and 24 hours at 37°C. After the right incubation time the cells were harvested and washed with cold PBS. After the wash the PBS was discarded and the cell were fixed using ethanol 70% and stored at -20°C for 24 hours. After the fixation period the ethanol was removed by one wash in PBS and the cells were incubated for 30 minutes at 37°C with the staining mix (sodium citrate pH 2.5 100mM, propidium iodide 2.5mg/ml, RNAse 10mg/ml and dH2O). The cell cycle analysis was conducted using the BD FACS Canto and the quantitative analysis using Modfit LT software (Verity).

### Phospho-histone H3/propidium Iodide co-staining

To evaluate the expression of phospho-Histone H3 (marker of mitosis) different cell lines were treated with prexasertib (IC50 after 24 hours). The cell lines were seeded at the concentration of 500,000 cell/ml and treated for 18, 24, 30 and 48 hours at 37°C. After the right period of incubation the cell were harvested, washed twice in ice cold PBS and fixed in -20°C with 70% ETOH for 24 hours. After the right fixation period the cells were washed twice with PBS+0.5%Tween 20 and with PBS+0.1% BSA. The cells were incubated in darkness with FITC-conjugated Phospho-HH3 antibody, dilution rate 1:100 in PBS+0.1%BSA, for 1 hour on ice (Phospho-Histone H3 (ser10) Antibody Alexa Fluor 488 conjugate #9708 Cell Signaling).To remove the exceeded of antibody the samples were washed twice in PBS and the fixed for 30 minutes with -20°C 70%ETOH. After the second fixation the cells were washed twice in cold PBS and then stained with the staining mix(( sodium citrate pH 2.5 100mM, propidium iodide 2.5mg/ml, RNAse 10mg/ml and dH2O) for 30 minutes at room temperature. The cell cycle analysis and the detection of the phosphor-HH3 positive cells were conducted using the BD FACS Canto and the quantitative analysis using Modfit LT software (Verity).

### Statistics

All the differences in percentages of reduction of the cell viability were analyzed by unpaired *t*-test (P≤0.05 was considered as statistically significant).

## SUPPLEMENTARY FIGURE



## References

[1] Moorman A V, Moorman A (2015). New and emerging prognostic and predictive genetic biomarkers in B-cell precursor acute lymphoblastic leukemia. Hematology Am Soc Hematol Educ Program.

[2] Foa R, Vitale A, Vignetti M, Meloni G, Guarini A, Propris MS De (2011). Dasatinib as first-line treatment for adult patients with Philadelphia chromosome – positive acute lymphoblastic leukemia. Therapy.

[3] Vignetti M, Fazi P, Cimino G, Martinelli G, Di Raimondo F, Ferrara F (2007). Imatinib plus steroids induces complete remissions and prolonged survival in elderly Philadelphia chromosome-positive patients with acute lymphoblastic leukemia without additional chemotherapy: Results of the Gruppo Italiano Malattie Ematologiche dell'Adu. Blood.

[4] Leoni V, Biondi A (2015). Tyrosine kinase inhibitors in BCR-ABL positive acute lymphoblastic leukemia. Haematologica.

[5] Tamura K (2015). Development of cell-cycle checkpoint therapy for solid tumors. Japanese Journal of Clinical Oncology.

[6] Stewart ZA, Westfall MD, Pietenpol JA (2003). Cell-cycle dysregulation and anticancer therapy. Trends in Pharmacological Sciences.

[7] Benada J, Macurek L (2015). Targeting the Checkpoint to Kill Cancer Cells. Biomolecules.

[8] Kastan MB, Bartek J (2004). Cell-cycle checkpoints and cancer. Nature.

[9] Bartek J, Lukas J (2001). Pathways governing G1/S transition and their response to DNA damage. FEBS Lett.

[10] Sherr CJ, Roberts JM (1999). CDK inhibitors: positive and negative regulators of G1-phase progression. Genes Dev.

[11] Finn K, Lowndes NF, Grenon M (2012). Eukaryotic DNA damage checkpoint activation in response to double-strand breaks. Cell Mol Life Sci.

[12] Zhao H, Piwnica-Worms H (2001). ATR-mediated checkpoint pathways regulate phosphorylation and activation of human Chk1. Mol Cell Biol.

[13] Zhang Y, Hunter T (2014). Roles of Chk1 in cell biology and cancer therapy. Int J Cancer.

[14] Smith J, Mun Tho L, Xu N, Gillespie DA (2010). The ATM-Chk2 and ATR-Chk1 pathways in DNA damage signaling and cancer. Advances in Cancer Research.

[15] Patil M, Pabla N, Dong Z (2013). Checkpoint kinase 1 in DNA damage response and cell cycle regulation. Cell Mol Life Sci.

[16] Collins I, Garrett MD (2005). Targeting the cell division cycle in cancer: CDK and cell cycle checkpoint kinase inhibitors. Curr Opin Pharmacol.

[17] Garrett MD, Collins I (2011). Anticancer therapy with checkpoint inhibitors: What, where and when?. Trends Pharmacol Sci.

[18] McNeely S, Beckmann R, Bence Lin AK (2014). CHEK again: Revisiting the development of CHK1 inhibitors for cancer therapy. Pharmacol Ther.

[19] Dillon MT, Good JS, Harrington KJ (2014). Selective Targeting of the G2/M Cell Cycle Checkpoint to Improve the Therapeutic Index of Radiotherapy. Clin Oncol.

[20] Dent P, Tang Y, Yacoub A, Dai Y, Fisher PB, Grant S (2011). CHK1 inhibitors in combination chemotherapy: thinking beyond the cell cycle. Mol Interv.

[21] Derenzini E, Agostinelli C, Imbrogno E, Iacobucci I, Brighenti E, Righi S (2015). Constitutive activation of the DNA damage response pathway as a novel therapeutic target in diffuse large B-cell lymphoma. Oncotarget.

[22] Iacobucci I, Di Rorà AGL, Falzacappa MVV, Agostinelli C, Derenzini E, Ferrari A (2015). In vitro and in vivo single-agent efficacy of checkpoint kinase inhibition in acute lymphoblastic leukemia. J Hematol Oncol.

[23] Zabludoff SD, Deng C, Grondine MR, Sheehy AM, Ashwell S, Caleb BL (2008). AZD7762, a novel checkpoint kinase inhibitor, drives checkpoint abrogation and potentiates DNA-targeted therapies. Mol Cancer Ther.

[24] Thompson R, Eastman A (2013). The cancer therapeutic potential of Chk1 inhibitors: How mechanistic studies impact on clinical trial design. Br J Clin Pharmacol.

[25] Reader JC, Matthews TP, Klair S, Cheung KMJ, Scanlon J, Proisy N (2011). Structure-guided evolution of potent and selective CHK1 inhibitors through scaffold morphing. J Med Chem.

[26] King C, Diaz HB, McNeely S, Barnard D, Dempsey J, Blosser W (2015). LY2606368 causes replication catastrophe and anti-tumor effects through CHK1-dependent mechanisms. Mol Cancer Ther.

[27] Narayanan S, Shami PJ (2012). Treatment of acute lymphoblastic leukemia in adults. Critical Reviews in Oncology/Hematology.

[28] Information C, Information O (2016). Tyrosine kinase inhibitors in Ph + acute lymphoblastic leukaemia : facts and perspectives. Ann Hematol.

[29] Majda K, Lubecka K, Kaufman-Szymczyk A, Fabianowska-Majewska K (2011). Clofarabine (2-chloro-2???-fluoro-2???-deoxyarabinosyladenine) - Biochemical aspects of anticancer activity. Acta Pol Pharm - Drug Res.

[30] Barba P, Sampol A, Calbacho M, Gonzalez J, Serrano J, Mart??nez-S??nchez P (2012). Clofarabine-based chemotherapy for relapsed/refractory adult acute lymphoblastic leukemia and lymphoblastic lymphoma. The Spanish experience. American Journal of Hematology.

[31] Lech-Maranda E, Korycka A, Robak T (2009). Clofarabine as a novel nucleoside analogue approved to treat patients with haematological malignancies: mechanism of action and clinical activity. Mini Rev Med Chem.

[32] Xiao Y, Ramiscal J, Kowanetz K, Del Nagro C, Malek S, Evangelista M (2013). Identification of Preferred Chemotherapeutics for Combining with a CHK1 Inhibitor. Mol Cancer Ther.

[33] Nguyen T, Hawkins E, Kolluri A, Kmieciak M, Park H, Lin H (2015). Synergism between bosutinib (SKI-606) and the Chk1 inhibitor (PF-00477736) in highly imatinib-resistant BCR/ABL+ leukemia cells. Leuk Res.

[34] Beeharry N, Banina E, Hittle J, Skobeleva N, Khazak V, Deacon S (2014). Re-purposing clinical kinase inhibitors to enhance chemosensitivity by overriding checkpoints. Cell Cycle.

[35] Kim MK, James J, Annunziata CM (2015). Topotecan synergizes with CHEK1 (CHK1) inhibitor to induce apoptosis in ovarian cancer cells. BMC Cancer.

[36] Wang FZ, Fei HR, Cui YJ, Sun YK, Li ZM, Wang XY (2014). The checkpoint 1 kinase inhibitor LY2603618 induces cell cycle arrest, DNA damage response and autophagy in cancer cells. Apoptosis.

[37] Bryant C, Scriven K, Massey AJ (2014). Inhibition of the checkpoint kinase Chk1 induces DNA damage and cell death in human Leukemia and Lymphoma cells. Mol Cancer.

[38] Bryant C, Stokes S, Massey a (2011). J. Abstract 4458: Chk1 inhibition as a novel therapeutic strategy for treating triple negative breast and ovarian cancers. Cancer Res.

[39] Seedhouse C, Grundy M, Shang S, Ronan J, Pimblett H, Russell N (2009). Impaired S-phase arrest in acute myeloid leukemia cells with a FLT3 internal tandem duplication treated with clofarabine. Clin Cancer Res.

[40] Nieborowska-Skorska M, Stoklosa T, Datta M, Czechowska A, Rink L, Slupianek A (2006). ATR-Chk1 axis protects BCR/ABL leukemia cells from the lethal effect of DNA double-strand breaks. Cell Cycle.

[41] Sarmento LM, Póvoa V, Nascimento R, Real G, Antunes I, Martins LR (2014). CHK1 overexpression in T-cell acute lymphoblastic leukemia is essential for proliferation and survival by preventing excessive replication stress. Oncogene.

