# Supplementary material for: Prexasertib, a Chk1/Chk2 inhibitor, increases the effectiveness of conventional therapy in B-/T- cell progenitor acute lymphoblastic leukemia
Source: Oncotarget. 2016 Jul 11;7(33):53377–91. doi: 10.18632/oncotarget.10535 (PMC5288194; doi:10.18632/oncotarget.10535)
Supplement: Supplementary file 1 [file oncotarget-07-53377-s001.pdf]

Prexasertib, a Chk1/Chk2 inhibitor, increases the effectiveness of conventional therapy in B-/T- cell progenitor acute lymphoblastic leukemia

SUPPLEMENTARY FIGURE

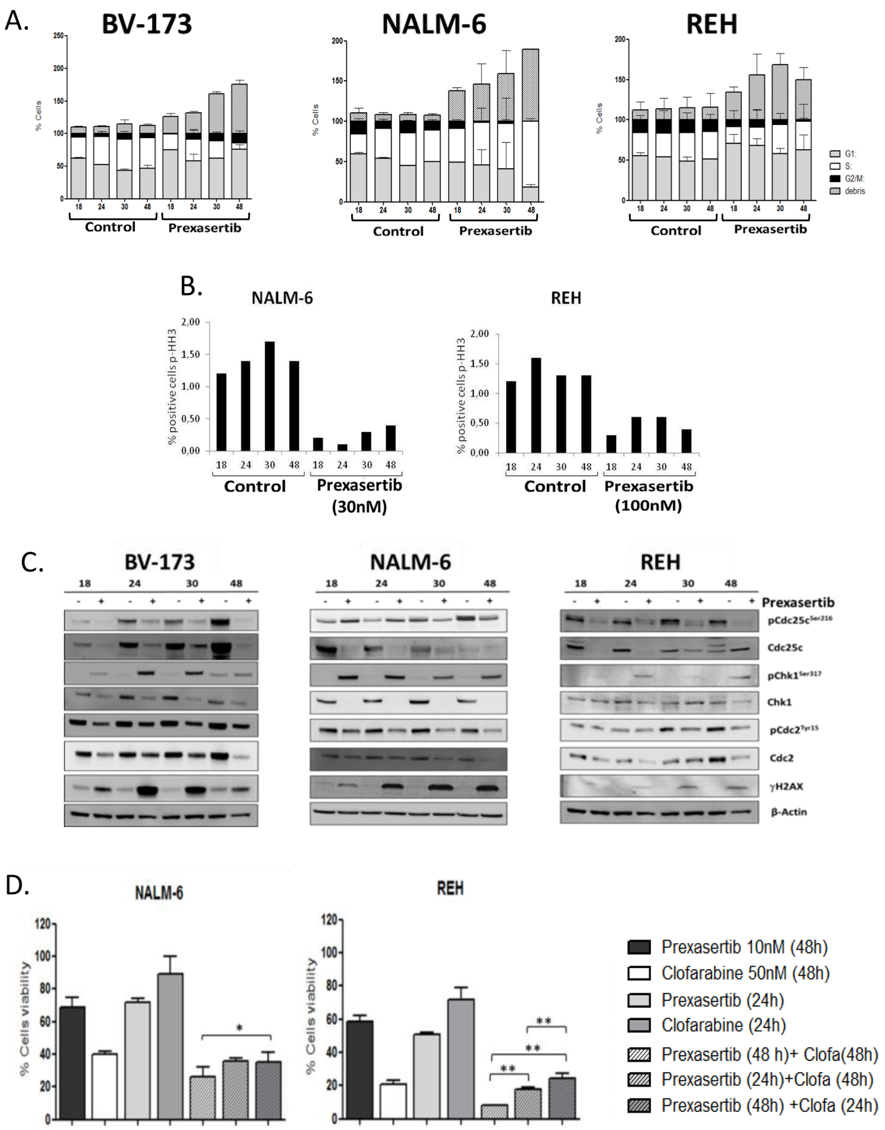

Supplementary Figure S1: Cell cycle profile of BV-173, NALM-6 and REH after 18, 24, 30 and 48 hours of incubation with prexasertib (IC_{50} value) A. Schematic representation of the number of cells positive for phospho-HH3 (ser10) antibody as marker of mitosis. NALM-6 and REH cell lines were incubated with or without prexasertib (30 and 100 nM respectively) for 18, 24, 30 and 48 hours. In the graph the amount of positive cells for Phospho-HH3(Ser10) are showed as a percentage of the total amount of cells **B.** The blots show the expression of different proteins of the Chk1 pathway on BV-173, NALM-6 and REH cell lines after 18, 24, 30 and 48 hours of incubation with prexasertib (IC50 value) **C.** Cell viability assay of NALM-6 and REH cell lines treated with prexasertib and clofarabine using different schedules **D.**
